# Implementation and expansion of laboratory capacity for molecular diagnostics in response to COVID‐19 and preparedness for other emerging infectious diseases in the Islamic Emirate of Afghanistan

**DOI:** 10.1111/irv.13210

**Published:** 2023-11-12

**Authors:** Murad Momin Khan, Mohamed Mostafa Tahoun, Luke W. Meredith, Amal Barakat, Hafizullah Safi, Ahmed Nasir Hanifi, Mohammad Omar Mashal, Abdul Wahid Amiri, Alaa Abouzeid

**Affiliations:** ^1^ Afghanistan Country Office World Health Organization Kabul Afghanistan; ^2^ High Institute of Public Health Alexandria University Alexandria Egypt; ^3^ Eastern Mediterranean Regional Office World Health Organization Cairo Egypt; ^4^ Directorate of Diagnostic Services Ministry of Public Health Kabul Afghanistan; ^5^ Faculty of Medicine Cairo University Cairo Egypt

**Keywords:** Covid‐19 pandemic, decentralized diagnostics, emerging diseases, genomic surveillance, outbreaks

## Abstract

**Background:**

Afghanistan experienced various outbreaks before and during the Covid‐19 pandemic, including dengue, Crimean Congo hemorrhagic fever (CCHF), measles, and acute watery diarrhea (AWD). Diagnostic and surveillance support was limited, with only the Central Public Health Laboratory equipped to handle outbreak responses. This article highlights initiatives taken to improve diagnostic capabilities for COVID‐19 and other outbreaks of public health concern encountered during the pandemic.

**Background:**

The World Health Organization (WHO) Afghanistan Country Office collaborated with the WHO Eastern Mediterranean Regional Office (EMRO), Central Public Health Laboratory (CPHL), and National Influenza Center (NIC) to enhance COVID‐19 diagnostic capacity at national and subnational facilities. To alleviate pressure on CPHL, a state‐of‐the‐art laboratory was established at the National Infectious Disease Hospital (NIDH) in Kabul in 2021–2022, while WHO EMRO facilitated the regionalization of testing to subnational facilities for dengue, CCHF, and AWD in 2022–2023.

**Results:**

COVID‐19 testing capacity expanded nationwide to 34 Biosafety Level II labs, improving diagnosis time. Daily testing rose from 1000 in 2020 to 9200 in 2023, with 848,799 cumulative tests. NIDH identified 229 CCHF cases and 45 cases nationally. Dengue and CCHF testing, decentralized to Nangarhar and Kandahar labs, identified 338 dengue and 18 CCHF cases. AWD testing shifted to NIDH and five subnational facilities (Kandahar, Paktia, Balkh, Herat, and Nangarhar labs), while measles testing also decentralized to nine subnational facilities.

**Conclusion:**

Afghanistan implemented a remarkable, multisectoral response to priority pathogens. The nation now possesses diagnostic expertise at national and subnational levels, supported by genomic surveillance. Future efforts should concentrate on expanding and sustaining this capacity to enhance public health responses.

## 1 INTRODUCTION

**FIGURE 1:**
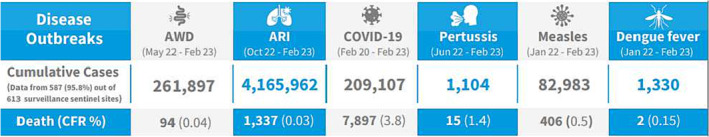
Overview of current outbreak situation in Afghanistan as at February 2023. The World Health Organization (WHO)/Eastern Mediterranean Regional Office (EMRO) has been monitoring four active outbreaks in Afghanistan, concurrent to the COVID‐19 outbreak. Pathogens including Congo hemorrhagic fever (CCHF), dengue, cholera, measles, and more were identified at outbreak levels, placing stress on testing and health infrastructure during the COVID‐19 pandemic.

The Islamic Emirate of Afghanistan, a landlocked country at the crossroads of Central Asia and South Asia, has been grappling with numerous challenges including civil discord, chronic poverty, drought, food insecurity, and recurring disease outbreaks.^1^ These issues have been exacerbated by acute economic and political turmoil since 2012, resulting in one of the world's worst humanitarian crises. Consequently, the health system has faced immense pressure to address the escalating health needs of the population of 40.1 million people. Over the past 3 years, Afghanistan has experienced multiple outbreaks, including dengue virus, measles, Pertussis and more recently, Crimean Congo hemorrhagic fever (CCHF), and acute watery diarrhea (AWD) (Figure 1).^1^ The limited testing capacity at diagnostic laboratories has been severely strained by the surging number of COVID‐19 cases, impacting over 800,000 people in the country thus far.^1^

Before the COVID‐19 pandemic, the Central Public Health Laboratory (CPHL) was the sole facility equipped to handle novel pathogen outbreaks, while regional reference laboratories focused on diagnosing measles, rubella, and rotavirus. The CPHL possessed the necessary capacity to conduct polymerase chain reaction (PCR), enzyme‐linked immunosorbent assay (ELISA), influenza virus and bacterial culture, and other diagnostic techniques. Its experienced staff played a crucial role in diagnosing both endemic and outbreak diseases in the country. However, when the first COVID‐19 case emerged in February 2020, the laboratory's resources were redirected to prioritize COVID‐19 diagnostics and testing, leading to severe limitations in testing for other pathogens despite ongoing outbreaks in the region.

With support from the World Health Organization (WHO), Afghanistan implemented a scale‐up plan that significantly expanded the capacity for PCR and routine diagnostic screening of COVID‐19. However, the country faced limitations in its ability to identify variants and assess vaccine response due to the lack of widespread genomic capacity. Genomic surveillance has proven crucial in various scenarios, as it provides vital information to guide public health responses and allocate resources effectively.^2^, ^3^ Unfortunately, logistical and political challenges restricted the option of sending samples out of the country for genomic analysis or testing support. As a result, there was limited knowledge regarding the transmission of COVID‐19, its variants, and other pathogens between Afghanistan and other countries.

The WHO/Eastern Mediterranean Regional Office (EMRO) acknowledged the challenges posed by exporting COVID‐19 testing samples, limited testing capacity, and the risk of neglecting other disease outbreaks due to resource constraints. To address these issues in Afghanistan, WHO developed a plan to enhance diagnostic capabilities. This involved leveraging existing infrastructure like the CPHL and National Influenza Center (NIC) while extending diagnostic services to subnational laboratories for comprehensive coverage. Additionally, the severity of other outbreaks necessitated expanding overall laboratory capacity, including a dedicated facility for testing and sequencing non‐COVID‐19 pathogens.

The increased capacity has enabled Afghanistan to significantly expand its COVID‐19 response while simultaneously monitoring other outbreaks. Additional support is now encouraged to enhance the country's expertise in testing and genomics for various pathogens. It is also important to align the operations of WHO‐funded hospital laboratories with the CPHL to ensure the long‐term sustainability of a comprehensive diagnostic network throughout the country.

## 2 BACKGROUND

### 2.1 Upscaling of diagnostic capacity for COVID‐19 from the CPHL to subnational laboratories

During the initial stages of the pandemic, Afghanistan faced limited diagnostic capacity for COVID‐19. To address this, WHO/EMRO facilitated the transfer of samples to the Clinical Virology Unit in Rotterdam, Netherlands, for diagnostics and sequencing assistance.^4^ However, this approach proved to be neither efficient nor cost‐effective. In February 2020, a meeting was convened to explore how WHO/EMRO could assist with COVID‐19 diagnosis in Afghanistan. The NIC at the CPHL emerged as a suitable facility with molecular diagnostic capabilities and adherence to biosafety requirements for respiratory pathogens.^5^

Funding from the World Bank, European Union (EU), Japan International Cooperation Agency (JICA), and United States Agency for International Development (USAID) facilitated logistical support for procurement and supply of COVID‐19 diagnostic kits and consumables. Sample collection and testing strategies were established in line with WHO guidelines, in collaboration with WHO/EMRO and the US Centers for Disease Control and Prevention (CDC). Additionally, a sample transport network was established in collaboration with the Ministry of Public Health to ensure timely delivery of samples to the laboratory. This was accomplished by recruiting a network of couriers, both private and public, to collect samples from collection stations, then transport them with appropriate triple packaging to the testing laboratories where they would be received and tested as soon as possible.

**FIGURE 2:**
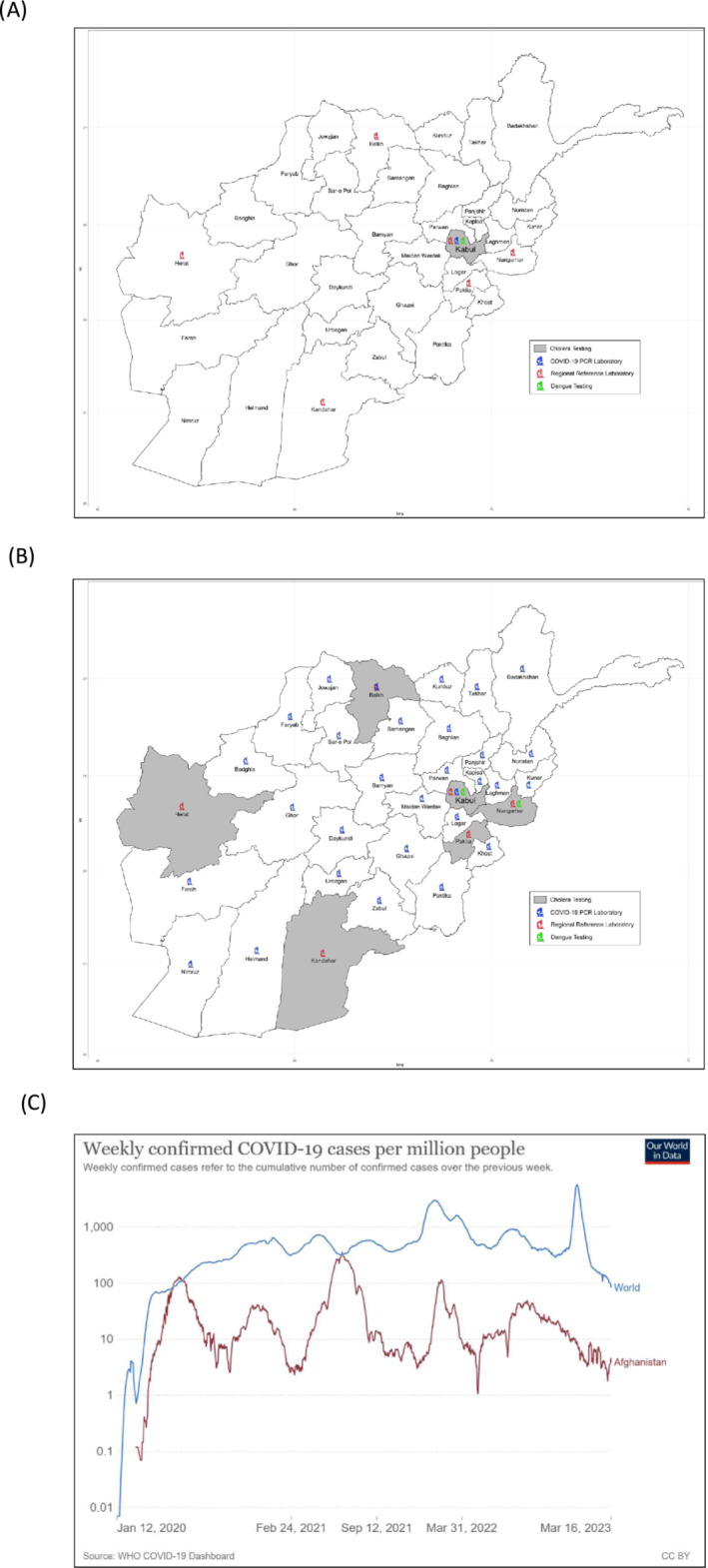
Afghanistan has implemented national molecular testing for COVID‐19, covering 100% of the population. (A) Initial molecular testing for COVID‐19 was carried out in the National Public Health Laboratory/National Influenza Centre only, resulting in delays in testing and results reporting. The World Health Organization (WHO)/Eastern Mediterranean Regional Office (EMRO), with support from stakeholders and the national Ministry of Public Health, provided logistical and technical support and training between January and April 2020 to support operationalization of decentralized testing in five regional reference hubs in March 2020, before rolling out further to 28 provincial testing laboratories. (B) Initial molecular diagnostic capacity was in Kabul (NPHL/National Influenza Center [NIC]) before expanding to regional and subnational laboratories, providing coverage to 99% of the population. (C) Molecular testing has identified five waves of infection in Afghanistan since 2020, which broadly coincide with international surges in infection (within 4 weeks of peaks of infection globally).

WHO/EMRO, in collaboration with international stakeholders, provided crucial support in training 150 national laboratory staff to ensure the sustainable and scalable delivery of laboratory services across Afghanistan. Testing began in five subnational reference laboratories between March and April 2020 (Figure 2). Subsequently, an additional 28 provincial PCR laboratories were established to ensure a decentralized network capable of timely COVID‐19 diagnosis. The WHO and Ministry of Public Health personnel facilitated comprehensive training programs, including central training courses at the CPHL and on‐site training at regional reference laboratories. These courses covered all aspects of reliable and reproducible COVID‐19 diagnostics using molecular techniques (Table 1).

**TABLE 1:** Training courses were provided to expand national expertise in molecular detection of COVID‐19 at the national and subnational level.

	Training course	Location	Dates	Topics covered
**1**	Laboratory diagnosis of COVID‐19 by RT‐PCR	CPHL‐Kabul	March 20	PPE use, RNA extraction techniques, programing PCR machine, result interpretation and reporting
**2**	Nasopharyngeal and oropharyngeal sample collection and processing on PCR	Afghan Japan Hospital Kabul	July 20	Techniques of sample collection, packaging, transportation, and processing on PCR
**3**	Diagnosis of COVID‐19 by RT‐PCR and reporting to the surveillance	Ibn‐e‐Sina Hospital, Kabul	October 20	PPE use, RNA extraction techniques, programing PCR machine, result interpretation and reporting
**4**	Diagnosis of COVID‐19 by RT‐PCR	Ghazanfar Institute, Kabul	February 21	GLP in laboratory, maintenance of lab equipment, and performing COVID‐19 sample testing on PCR
**5**	COVID‐19 sample collection, transportation, and processing by PCR	Ghazanfar Institute, Kabul	January 22	Techniques of sample collection, packaging, transportation, and processing on PCR
**6**	Laboratory diagnosis of COVID‐19 by RT‐PCR	Ghazanfar Institute, Kabul	March 22	GLP in laboratory, maintenance of lab equipment, and performing COVID‐19 sample testing on PCR
**7**	Impact of sample quality on PCR and Genomic sequencing results.	CPHL‐Kabul	March 23	Sample selection criteria, aliquots preparation, labeling, metadata file updating, sample storage, packaging and transportation following IATA rule and regulations.

*Note*: Staff from the CPHL and NIC received hands‐on training in laboratory testing for COVID‐19 from national and international partners, using a “train‐the‐trainers” approach. Training was then rolled out to subnational laboratories, initially to five regional reference laboratories before being rolled out to a further 28 provincial PCR laboratories. Courses were designed to ensure safe and reproducible molecular testing.

### 2.2 Support and operationalization of genomic surveillance for COVID‐19 and influenza

In mid‐late 2022, with financial support from WHO, CPHL established genomic sequencing for COVID‐19. This was made possible by the provision of a third generation sequencing analyzer (Oxford Nanopore Technology), as well as the necessary reagents and consumables. Additionally, WHO/EMRO and collaborators offered online bioinformatics support to assist with data analysis. For sustainability, supported by WHO/EMRO and the US CDC, the laboratory staff underwent two international training programs. The first training focused on genomic sequencing of SARS‐COV‐2 and took place at the Sheik Khalifa Medical Complex in Abu Dhabi in December 2021. The second training, held in November 2022 at the CPHL in Muscat, Oman, covered genomic sequencing of influenza A virus and the spike protein of SARS‐COV‐2.

The training courses conducted by international experts encompassed various essential topics, including sample collection and preparation, sequencing library preparation utilizing the Midnight SARS‐CoV‐2 workflow from Oxford Nanopore Technologies,^6^, ^7^ sequencing techniques, basic bioinformatics, and data analysis. As a result, national staff members are now equipped with the necessary skills to carry out genomic sequencing testing. The generated data are shared with WHO/EMRO staff to facilitate more comprehensive analysis and integration into reports. Ongoing efforts are being made to obtain permission and support for enrolling and depositing the data directly into international databases such as GISAID, which are publicly accessible and serve as valuable resources for global research and surveillance purposes.

### 2.3 Expansion of molecular and laboratory diagnostics for pathogens other than COVID‐19

Due to the increased demand for molecular testing related to COVID‐19, the CPHL NIC had to allocate a significant portion of capacity to SARS‐CoV‐2 testing. However, it was crucial to ensure support for other priority pathogens and ongoing outbreaks in Afghanistan. In response, WHO/EMRO facilitated the establishment of an advanced laboratory at the National Infectious Disease Hospital (NIDH) in Kabul. The primary objective of this laboratory was to provide molecular diagnostic support for priority arboviral and waterborne pathogens that were already circulating in the country before the COVID‐19 pandemic.

The NIDH advanced laboratory was equipped with PCR, GeneXpert, and serological capacity to detect diseases such as dengue fever, CCHF, and other hemorrhagic fevers, as well as waterborne pathogens like cholera (Table 2). Staff members received training from WHO/EMRO and the Ministry of Public Health, specifically from the CPHL, and began receiving samples for testing in early 2022. Operating as a national reference center, the laboratory receives samples from hospitals across the country for testing in cases of acute infections that meet the defined criteria outlined in WHO guidelines.^5^

**TABLE 2:** The advance lab at NIDH, Kabul, has testing capacity for a broad range of arboviral, hepatitis, and waterborne pathogens.

	Molecular detection (PCR/GeneXpert)	ELISA detection	Bacterial culture
**1**	CCHF virus	CCHF (Ag, IgM, IgG)	Cholera
**2**	Dengue (Type I‐IV)	Dengue (Ag, IgM, IgG)	
**3**	Hepatitis A virus	Hepatitis A virus (IgM, IgG)	
**4**	Hepatitis B virus	Hepatitis E virus (IgM, IgG)	
**5**	Hepatitis C virus		
**6**	Hepatitis E virus		

*Note*: The laboratory is equipped with multiple RT‐PCR machines, GeneXpert, ELISA capacity, and bacterial culture facilities, supporting testing of up to 250 samples of each pathogen, though equipment limitations mean that the total number of samples processed at any given time is 250 in total.

## 3 OUTCOME

### 3.1 COVID‐19 capacity expanded from central testing at CPHL to regional and provincial laboratories, providing coverage to the whole 34 provinces of the country

At the declaration of the Covid‐19 pandemic, CPHL had the capacity to support COVID‐19 diagnostics through PCR testing at Biosafety level II (BSL2) laboratories. The WHO supported training courses for CPHL and national staff to expand this capacity using a “train‐the‐trainer” approach, allowing for further expansion of testing across the country. By mid‐2022, 34 Biosafety level II laboratories were testing COVID‐19, and this capacity continues to be available for operation today. Two labs are in Central Kabul, (including CPHL): 5 are regional reference labs and 28 are provincial PCR labs (Figure 2A,B). All the labs are supported by the WHO/EMRO and the NIC. They are also provided with trained personnel and equipment like PCR machines, Freezers, Centrifuges, UPS system, diagnostic kits, and ancillary reagents. These laboratories were also enrolled in the WHO External Quality Assurance program for SARS‐CoV‐2 detection to ensure that testing was of the highest standard.

As of September 17, 2022, Afghanistan had 35 functioning COVID‐19 testing laboratories distributed across 34 provinces. The combined testing capacity for COVID‐19 was estimated to be 9200 tests per day. Additionally, near point‐of‐care testing was available in 12 provinces using the GeneXpert platform, providing an additional capacity of 1000 tests per day. Consequently, Afghanistan had a national molecular diagnostic capacity of 10,200 tests per day.

From the beginning of the pandemic until May 20, 2023, Afghanistan conducted testing on 848,799 samples for COVID‐19. Among these samples, 220,545 cases were confirmed positive, resulting in a cumulative positivity rate of 26.0%.

The expanded diagnostic capacity has allowed Afghanistan to monitor multiple waves of infection. Notably, five waves of infection were identified in June and December of each year between 2020 and 2022 (Figure 2C). These peaks generally aligned with international infection peaks,^8^ suggesting a potential association with seasonal or travel factors. Global studies are currently ongoing to determine the precise causes of these infection waves.

### 3.2 Implementation of genomic surveillance using the Oxford Nanopore Technology platform identifies omicron as the dominant variant circulating in Afghanistan

Genomic surveillance plays a crucial role in tracking circulating variants and detecting the emergence of new or novel variants within a population. It provides valuable insights into the effectiveness of public health interventions, including diagnostics and vaccination efforts.^2^, ^9^ Recognizing its significance, WHO/EMRO supported the implementation of COVID‐19 genomics in Afghanistan.

In order to establish in‐country genomics capabilities, WHO/EMRO supplied CPHL in Afghanistan with the Oxford Nanopore Technology MinION platform. This platform, accompanied by ancillary reagents and bioinformatics support, enabled the genomic sequencing of COVID‐19 samples within the country. The selection of the Nanopore technology was driven by its advantages, including its portability, robustness, and simplified library preparation process, as well as the streamlined workflows it offers for bioinformatics analysis.^6^, ^7^ Moreover, the availability of reliable and long‐lasting consumables associated with this technology contributes to the long‐term sustainability of genomics activities in Afghanistan.

The operationalization of the genomic sequencing capacity in Afghanistan took place in June 2022. The initial batch of 23 samples revealed that the Omicron variant was the predominant circulating variant, accounting for 69.23% (16/23) of the samples sequenced (Table 3). Subsequently, an additional eight samples were sequenced in the following months, with the dominant variant being 19A. However, it is important to note that the average turnaround time for obtaining sequencing results is currently high, standing at 3 months, though only two sequencing runs were carried out in the time period covered by this article due to logistical challenges receiving reagents. This delay is anticipated to improve with additional training and enhanced logistical support to expedite transportation of samples to the laboratory for testing, as well as more frequent sequencing runs.

**TABLE 3:** Genomic surveillance identifies omicron as the dominant variant circulating in Afghanistan in late 2022/2023.

	Sequencing run date	Runs	Samples	Success	Failure	Rate	Variants
**1**	June 2022	2	14	14	0	100%	Omicron (9), 19A (5)
			29	9	20	31%	Omicron (7), 20B (2)
**2**	January 2023	1	32	8	22	25%	Omicron (1), 19A (4), Recombinant (3)

*Note*: A total of 75 samples were sequenced using the Oxford Nanopore Technology Midnight Protocol, with data being analyzed through the EPI2ME SARS‐CoV‐2 pipeline, and variants assigned using the Pangolin online platform. Success rate for sequencing has been low, due to sample transport and storage issues, which will be addressed before further genomics runs are continued.

The logistical challenges encountered during sample transportation may partially account for the suboptimal quality of the sequencing results obtained thus far. Sample degradation can occur during transport, affecting the quality of the results obtained. Currently, the success rate for sequencing is relatively low, with only 41% of samples achieving full genome coverage. Notably, most successful samples were obtained during the initial run under the guidance of international experts.

To enhance the quality of results, national staff members have been closely collaborating with international stakeholders to provide further training courses and hands‐on support. Their collective efforts aim to improve the success rate of sequencing and enhance the quality of the obtained genomes. Additionally, retrospective sharing of further genomes is anticipated as part of ongoing efforts. Efforts are being made to incorporate sequencing of other pathogens of epidemic or pandemic concern, such as arboviruses, measles virus, viral hemorrhagic fever viruses, into the national surveillance program, though this will be a stepwise process over the medium to long term, as capacity at this stage is limited by the resources and expertise available to the country.

### 3.3 Provision of extra laboratory capacity for continued surveillance of nonrespiratory pathogens in a resource‐limited system

Due to the significant impact and global spread of COVID‐19, resources and expertise were primarily directed towards the CPHL to prioritize testing for SARS‐CoV‐2. This allocation of resources aimed to prevent the undetected transmission of a respiratory virus within communities. Consequently, the capacity to monitor and survey other pathogens such as dengue and CCHF, which have a significant impact on public and personal health, was limited.

Acknowledging the importance of continuing surveillance for these pathogens, the WHO/EMRO supported the operationalization of an advanced laboratory at NIDH in Kabul. The primary objective of this advanced lab was to ensure the ongoing surveillance of dengue and CCHF, both of which are known to circulate within the country. This initiative aimed to maintain vigilance and monitoring of these pathogens, despite the prioritization of resources for COVID‐19 testing.

The advanced lab at NIDH has four full‐time staff with expertise in molecular diagnostics, serology, and bacterial culture, with the capacity to test 250 samples per week for each priority pathogen with multiple techniques, though reagent and equipment limitations mean that testing of 250 samples of one pathogen at a time is optimal (Table 3). Since its operationalization in January 2022, the advanced laboratory at the NIDH in Kabul has played a crucial role in identifying cases of highly pathogenic CCHF and dengue virus within Afghanistan.

**FIGURE 3:**
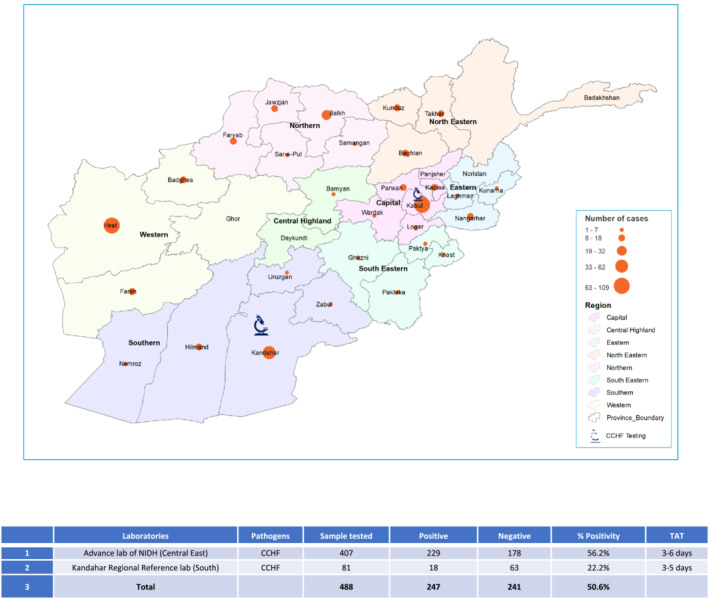
Molecular testing identified outbreaks of Congo hemorrhagic fever (CCHF) in regional Afghanistan during 2022–2023. Samples meeting case definition were collected from around the country as indicated by the map, then sent by courier to either the advance lab of the National Infectious Disease Hospital (NIDH) in Kabul or the Kandahar Regional Reference Laboratory for testing using polymerase chain reaction (PCR) for CCHF. Regions are indicated by color as per the figure legend, while regional case numbers are indicated by the size of the orange dots on the map. National results were compiled and tabulated, with turnaround time (TAT) calculated from the time the sample was collected and the results returned to the testing facility.

**FIGURE 4:**
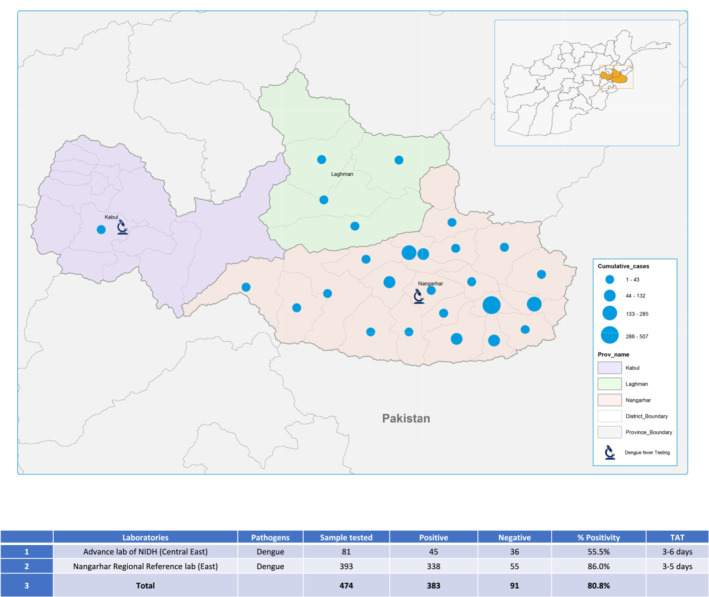
Decentralized testing of dengue virus at the Nangarhar Regional Reference laboratory identified high frequency of positive cases in symptomatic patients in Southern Afghanistan. Samples meeting case definition for dengue fever were collected from sites around the region as indicated by the map, then sent by courier to either the advance lab of the National Infectious Disease Hospital (NIDH) in Kabul or the Nangarhar Regional Reference Laboratory for testing using serology for dengue virus IgM. Provinces are indicated by color while the case number per region is indicated by the blue dots. Regional results were compiled, tabulated, and turnaround time (TAT) calculated from the time the sample was collected and the results returned to the testing facility.

To date, the laboratory has detected 229 cases of CCHF, caused by the highly pathogenic CCHF virus across various regions, including Central East, Central West, East, North, Northeast, Southeast, and West. Additionally, the laboratory has identified 45 cases of dengue fever caused by the dengue virus circulating in the Central East and East regions (Figures 3 and 4). Although these viruses have a lower level of transmissibility compared with SARS‐CoV‐2, they pose a significantly higher risk of mortality if left untreated. Therefore, timely detection and confirmation of diagnoses are critical in preventing the spread of these outbreaks. The advanced laboratory's role in detecting and identifying these cases is instrumental in preventing further transmission and effectively managing these potentially severe infections.

The identification of these cases triggered a public health response, with rapid response teams sent to support the communities in need. However, the logistical challenges associated with transporting samples even within the country have resulted in a need for further decentralization of testing. Decentralization of testing can be challenging for high‐containment pathogens such as cholera, measles, and dengue; however, in the context of clinical testing of suspected cases by PCR, there are clear guidelines for sample transport and handling, inactivation and extraction designed to protect both the laboratory staff and the community. These guidelines were key to the training and implementation of testing in the regional testing facilities.

### 3.4 Decentralization of testing in Afghanistan supported improved speed of response to dengue in the Eastern region of the country, capitalizing on investment in SARS‐CoV‐2 molecular detection

To address concerns about dengue detection in 2022, the sample testing capacity for both PCR and ELISA (IgM) was expanded to the Nangarhar Regional Reference Laboratory in the Eastern region of Afghanistan. This location was selected due to its proximity to the epicenter of dengue cases and its existing strong molecular and testing capabilities. The laboratory received facility upgrades and training in response to the COVID‐19 pandemic, including molecular methods training at CPHL, Kabul, in 2022. It has well‐trained technical staff proficient in molecular diagnostics, serology, and bacterial culture, making it an ideal facility for expanded testing. The laboratory now has the capacity to test 350 samples per week. The WHO provided support for capacity building training, covering safe sample collection, transportation, the use of Rapid Diagnostic Tests (RDTs), and laboratory diagnostic procedures.

Since its operationalization in June 2022, the Nangarhar Regional Reference has played a crucial role in timely responses to dengue outbreaks. The laboratory has successfully identified 338 positive cases of dengue in the Eastern region (Figure 4), allowing for immediate public health interventions. Compared with the CPHL and NIDH, the Nangarhar laboratory has a significantly faster turnaround time of 3 days for dengue sample testing (Figure 4). This is attributed to the reduced transport time to the facility.

The identification of these dengue cases has prompted a proactive public health response. Rapid support teams have been deployed to the affected communities, providing vital support through awareness campaigns, spray treatments, distribution of nets, and close monitoring of the outbreaks. These efforts aim to address the needs of the affected population and effectively mitigate the spread of the dengue virus.

### 3.5 Decentralization of acute watery diarrhea testing allowed continual monitoring of the ongoing cholera outbreak in Afghanistan

To address the continuous threat of AWD in Afghanistan, efforts were made to expand testing capacity beyond the CPHL in 2022. Due to the rapid increase in suspected cases during an AWD outbreak in 178 districts across all 34 provinces, resources in the CPHL were strained, necessitating the establishment of testing facilities in five regional reference laboratories (Kandahar, Paktia, Balkh, Herat, and Nangarhar).

To support this expansion, the Ministry of Public Health collaborated with WHO/EMRO and other stakeholders to conduct training courses on sample collection, transportation, processing, and diagnosis of AWD samples between June and July 2022. The WHO provided essential laboratory kits, including culture media, antisera, and consumables, to all the regional sites, ensuring their operational readiness.

**FIGURE 5:**
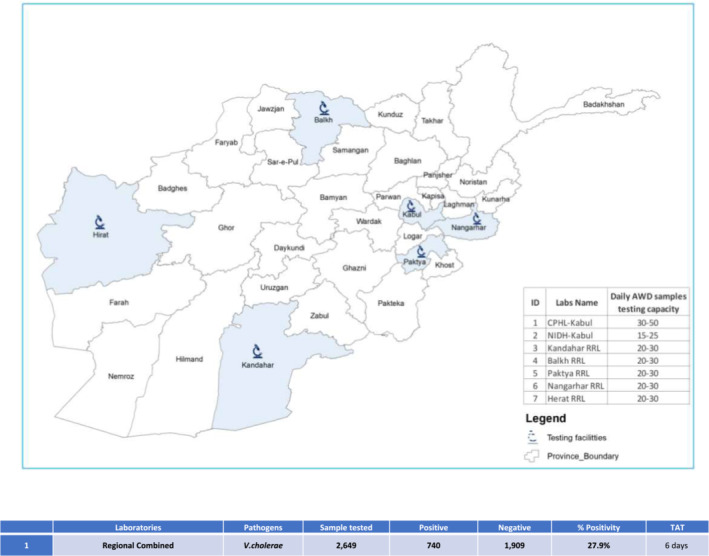
Decentralized testing of cholera identified high frequency of positive cases in symptomatic patients in Afghanistan. Samples collected from patients meeting case definition for AWD and specifically cholera were transported to the nearest regional reference laboratory for testing using culture‐based and serological assays. The testing capacity of each regional lab is indicated by the figure legend, while total positive cases, turnaround time (TAT), calculated form the time the sample was collected, are shown in the table.

Between May 1, 2022 and May 20, 2023, a total of 2649 samples for AWD were tested by the CPHL and regional reference laboratories (Figure 5). These samples were collected from various locations across the country and tested specifically for cholera as the samples met case definition. The positivity rate was found to be 27.9%. Notably, the implementation of decentralized testing reduced the turnaround time to an average of 6 days (Figure 4), enabling a more efficient and timely deployment of medical support and staff during the outbreak.

### 3.6 Seasonal outbreaks of measles remain a concern during the COVID‐19 pandemic

Measles remains a significant public health concern in Afghanistan, despite the availability of an effective vaccine.^10^ To address the increased demand for testing, CPHL decentralized measles testing to nine subnational sites. These sites include two in Central Kabul (Maiwand Hospital, Indra Ghandhi Hospital), five regional reference laboratories (Kandahar, Paktia, Nangarhar, Balkh, and Herat), and one located at the regional hospital of Kunduz province. All sites have the necessary serology capacity to test for measles antibodies (IgM/IgG) and confirm cases during outbreaks.

**FIGURE 6:**
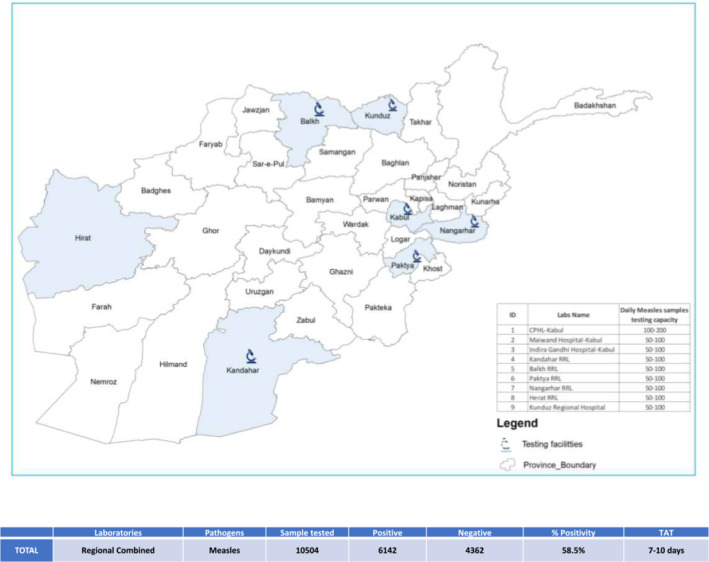
Decentralized testing of measles continues to identify a high frequency of positive cases in Afghanistan. Samples collected from patients nationally were transported to the nearest regional reference laboratories for testing using PCR and serology. Testing capacity in each regional lab is indicated on the figure, while case numbers, positivity, and turnaround time (TAT) are calculated form the time the sample was collected is indicated in the subsequent table.

Between January 2022 and May 20, 2023, a total of 12,682 samples were tested for measles in Afghanistan. Out of these, 7297 samples were confirmed positive in the laboratory, resulting in a positive ratio of 57.5%. The WHO provided support during this period by supplying ELISA kits, sample collection kits, consumables, and conducting refresher training for the staff. It is worth noting that the mean turnaround time for testing at CPHL during the COVID‐19 pandemic was 5–6 days (Figure 6), which was consistent with the turnaround time at the subnational level. This highlights the importance of having a robust and functional diagnostic network to ensure prompt detection and response to pathogens as they arise.

## 4 DISCUSSION

Thanks to the support of the WHO/EMRO, Afghanistan has achieved significant success in expanding COVID‐19 diagnostics throughout the country. Through the establishment of a decentralized network of 34 laboratories, including the CPHL, the country now has the capacity to conduct high‐quality and rapid turnaround COVID‐19 diagnostics. This expanded testing network allows for the timely detection and response to COVID‐19 outbreaks as they arise.

Furthermore, the implementation of genomics in Afghanistan has introduced a new dimension to disease surveillance. It has provided valuable insights into the circulating variants of the virus in the country and has the potential to assess the effectiveness of diagnostic tools, vaccine responses, and intervention strategies. This genomic surveillance platform will continue to inform public health decisions and guide future interventions in Afghanistan.

Implementing the response in Afghanistan faced significant challenges, including logistical delays due to the country's embargo and the need to identify alternative methods and suppliers. Sample transportation and training staff in remote areas were also logistically challenging, resulting in delays in establishing and operationalizing laboratories. These difficulties incurred higher costs compared with other countries, but the key lessons learned during the expansion, including the need for improved logistics and dissemination of reagents to support a subnational laboratory network, train‐the‐trainer approaches by international stakeholders, followed by disseminated learning nationally, and more can be applied in other countries with resource limitations. Indeed, decentralization of testing has allowed the country to identify outbreaks that previously may have been missed due to the lead time in transferring samples to the central laboratories.

The expansion of laboratory testing capacity in Afghanistan faced challenges in finding suitable spaces that met biosecurity and infrastructure requirements for molecular testing, particularly for pathogens with higher containment levels. Additionally, a stable power supply and internet access were necessary for the analysis and distribution of results in molecular testing. These challenges are expected to be encountered when expanding testing capacity for other pathogens as well. Understanding how these challenges were addressed during the expansion of COVID‐19 testing will help improve future responses.

In addition to the focus on COVID‐19, it was crucial to address the detection of other priority pathogens to prevent their neglect. The WHO/EMRO supported the establishment of an advanced diagnostic laboratory at the NIDH, which plays a key role in monitoring and detecting pathogens such as dengue and CCHF, ensuring prompt responses to outbreaks. The decentralization of testing for these pathogens to subnational facilities has further improved response times and can serve as a model for expanding testing capacity for other pathogens in the future. This comprehensive approach ensures that multiple priority pathogens are actively monitored and addressed alongside COVID‐19.

A significant challenge faced in Afghanistan's testing efforts is sample transport, primarily due to infrastructure limitations and inadequate equipment for maintaining sample quality during transportation. This poses a risk to the quality of samples received at national laboratories. To address this challenge, decentralization of testing is crucial. By expanding diagnostic capacity for COVID‐19 and enhancing expertise and capacity for nonrespiratory diseases, Afghanistan can establish a robust pathogen surveillance and response network. This presents a unique opportunity for sustained and expanded testing capabilities, enabling accurate and timely data for clinicians and guiding effective health responses across a broader range of diseases.

It should be noted, however, that challenges with continued funding, procurement, and logistics of importing reagents into the country are likely to limit the continued expansion and operation of the established network, particularly at the subnational level. The regional testing laboratories continue to be supported through the WHO/EMRO and the NIC program, but the provincial PCR testing laboratories have been wound back to a standby status due to a lack of resources for continued pathogen testing. This scale‐back of capacity is not unique to Afghanistan and indeed is being observed globally in response to the de‐classification of COVID‐19 as a PHEIC; however, it is important that these facilities be maintained for operational readiness in the case of emergence of new or re‐emerging pathogens of pandemic concern.

In addition to the ongoing commitment to funding and resourcing, sustaining the laboratory network will require that efforts be made to harmonize and train other labs, enhance genomic sequencing capacity, and ensure quality through EQA programs and professional development. This will create a resilient workforce capable of detecting various pathogens beyond COVID‐19, contributing to long‐term public health protection.

## 5 CONCLUSION

It is expected that Afghanistan will continue to face health challenges in future. Thus, investments made during the COVID‐19 pandemic in producing a highly functional, decentralized diagnostic laboratory network, while sustaining diagnostics for other pathogens, is a key success story from the pandemic and has produced a solid launching pad for future expansion of testing for other pathogens. Lessons learned regarding procurement and logistics, supply and technical training, travel, and transportation challenges should be consolidated to inform future responses to pandemics or outbreaks as they occur.

## AUTHOR CONTRIBUTIONS

M. M. K., M. T., H. S., A. H., M. M., and A. A. M. were responsible for data curation and analysis. All authors contributed to the drafts and reviews of the manuscript; L. W. M. is the corresponding author. A. B., A. H., and A. A. B. were responsible for the methodology, while A. B. and A. A. B. were responsible for administration and supervision of the project. A. A. B. was responsible for the conceptualization and delivery of the project.

## CONFLICT OF INTEREST STATEMENT

All authors declare no conflict of interest.

## Data Availability

Data included in this manuscript can be requested through the Afghanistan Ministry of Public Health, who maintain the final decision on release of data from the country.
